# A novel clip with mantis-like claws combined with the over-the-scope clip for fistula closure

**DOI:** 10.1055/a-2513-2683

**Published:** 2025-02-11

**Authors:** Koichiro Kawano, Mamoru Takenaka, Reiko Kawano, Daisuke Kagoshige, Koutaro Mine, Katsuhisa Nishi, Masatoshi Kudo

**Affiliations:** 1 Department of Gastroenterology, Hyogo Prefectural Awaji Medical Center, Sumoto, Japan; 2 Department of Gastroenterology and Hepatology, Kindai University Faculty of Medicine, Osakasayama, Japan

The over-the-scope (OTS) clip is a nitinol endoscopic clip widely used for hemostasis and fistula closure in the gastrointestinal tract 1 2 3 . During OTS clip application, endoscopic suction pulls tissue into the tip hood and the clip is released for fistula closure; however, suction alone may be insufficient due to inadequate traction. The Twin Grasper system (Ovesco Corp., Tübingen, Germany) is useful in such cases; it utilizes two jaws to grasp and pull tissue into the hood before releasing the clip. However, improper retraction can cause the OTS clip to bite into the Twin Grasper system, preventing its removal ( Fig. 1 **a, b** ) 4 . Thus, we developed a new technique to address this issue.

**Fig. 1:**
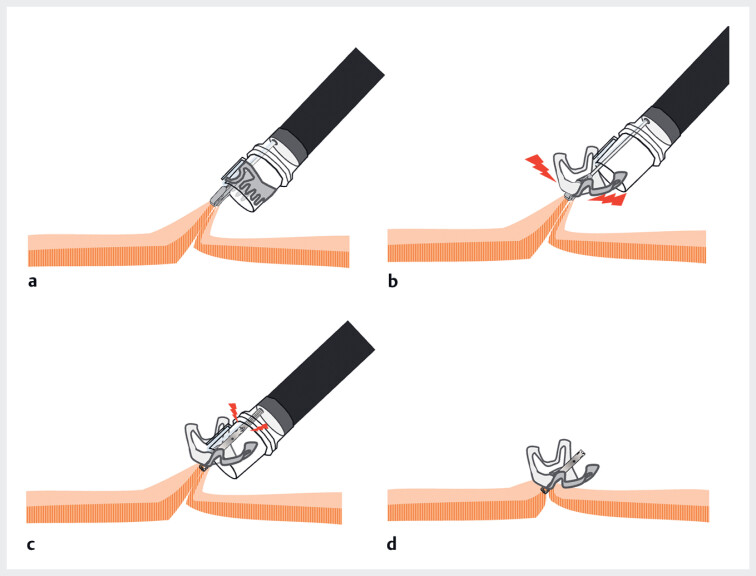
Over-the-scope (OTS) clip application. **a** The Twin Grasper system (Ovesco Corp., Tübingen, Germany) grasps both sides of the peristomal tissue and is retracted into the hood. **b** When the OTS clip is released with insufficient retraction, it may accidentally bite into the Twin Grasper system, making the removal of the device difficult. **c, d** If the OTS clip accidentally bites the MANTIS Clip, the device can be removed by releasing the MANTIS Clip.

The MANTIS Clip (Boston Scientific Japan, Tokyo, Japan), a novel clip with mantis-like claws, ensures improved tissue grasping and is useful for post-endoscopic submucosal dissection defect closure 5 . It offers excellent rotational performance and secure grasping ( Fig. 2 ), holding the perifistula tissue edges face-to-face ( Fig. 3 ).

**Fig. 2:**
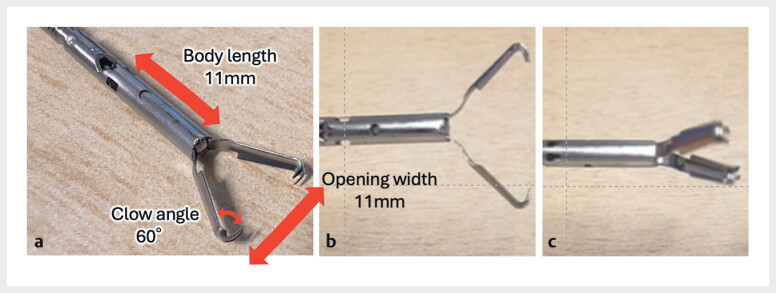
Features of the MANTIS Clip (Boston Scientific Japan, Tokyo, Japan). **a** MANTIS Clips have a 60° claw angle and are excellent at maintaining tissue grip. **b, c** MANTIS Clips are easy to rotate.

**Fig. 3:**
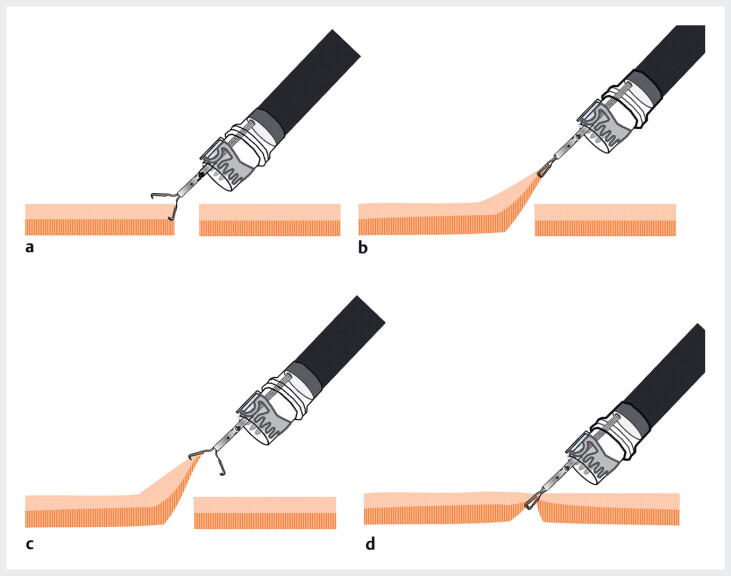
MANTIS Clip application (Boston Scientific Japan, Tokyo, Japan). **a** One edge of the perifistula tissue is grasped with the MANTIS Clip. **b** With the clip in place, the endoscope is guided to the opposite side of the fistula. **c** The MANTIS Clip is deployed, but its shape allows it to maintain its grip on the tissue. **d** The clip is closed, and the two edges of the perifistula tissue are grasped, facing each other.

A 70-year-old man underwent transanal total mesorectal excision for rectal cancer, and a fistula subsequently formed due to anastomotic failure ( Fig. 4 **a** ). Both tissue edges of the fistula were grasped using the MANTIS Clip ( Fig. 4 **b, c** ), and the OTS clip was released after sufficient traction, closing the fistula. Thereafter, the MANTIS Clip was released ( Fig. 5 ); the clip was dislodged due to tight tissue grasping, but the OTS clip successfully closed the fistula ( Fig. 4 **d** ; Video 1 ).

**Fig. 4:**
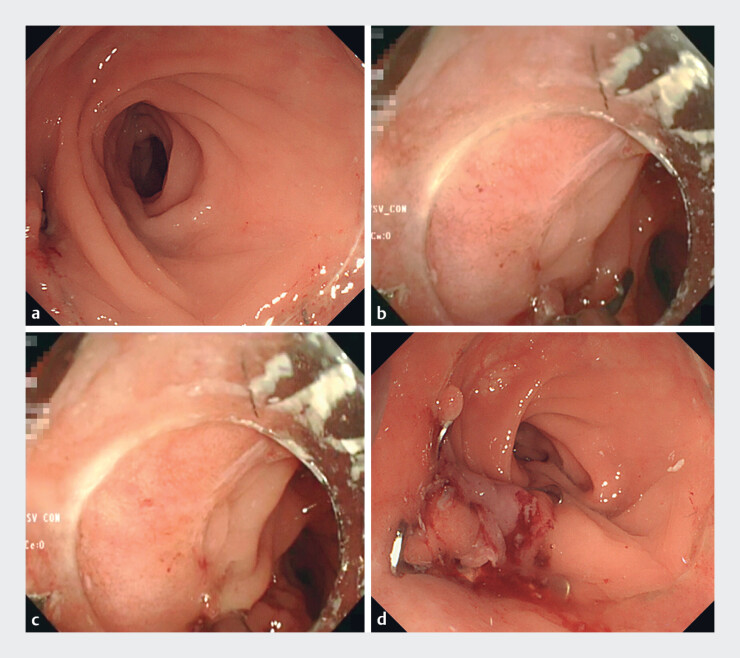
Endoscopic images of the fistula. **a** Fistula at the anastomosis. **b** After one edge of the fistula is grasped with the MANTIS clip (Boston Scientific Japan, Tokyo, Japan), the grasped tissue does not fall out of the clip when the MANTIS clip is deployed. **c** Both sides of the tissue are grasped with the MANTIS clip. **d** Fistula closed using the over-the-scope clip.

**Fig. 5:**
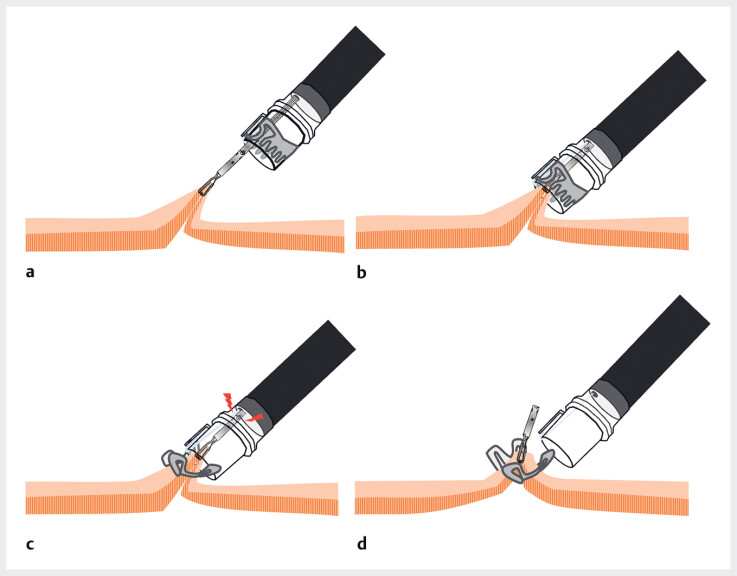
MANTIS Clip application (Boston Scientific Japan, Tokyo, Japan). **a, b** With both edges of the perifistula tissue fully grasped, the clip is retracted into the hood. **c** Following sufficient retraction, the over-the-scope clip is released, followed by the release of the MANTIS Clip. **d** Fistula closure is confirmed.

A new, cost-effective traction technique for safe grasping and tissue traction, resulting in successful fistula closure using the over-the-scope clip.Video 1

We described a simple, effective, and low-cost technique for reliable tissue grasping using the MANTIS Clip during fistula closure with the OTS clip. This technique has three advantages. First, the rotational performance of the MANTIS Clip allows precise orientation for tissue grasping. Second, releasing the MANTIS Clip poses no risk of device entrapment, even if the OTS clip bites into it. Finally, the MANTIS Clip is cost effective compared with other existing devices.

Endoscopy_UCTN_Code_TTT_1AQ_2AG

## Notes

Endoscopy E-Videos https://eref.thieme.de/e-videos
*E-Videos* is an open access online section of the journal *Endoscopy* , reporting on interesting cases and new techniques in gastroenterological endoscopy. All papers include a high-quality video and are published with a Creative Commons CC-BY license. Endoscopy E-Videos qualify for HINARI discounts and waivers and eligibility is automatically checked during the submission process. We grant 100% waivers to articles whose corresponding authors are based in Group A countries and 50% waivers to those who are based in Group B countries as classified by Research4Life (see: https://www.research4life.org/access/eligibility/ ). This section has its own submission website at https://mc.manuscriptcentral.com/e-videos .
